# Transcriptomic features of *Pecten maximus* oocyte quality and maturation

**DOI:** 10.1371/journal.pone.0172805

**Published:** 2017-03-02

**Authors:** Marianna Pauletto, Massimo Milan, Arnaud Huvet, Charlotte Corporeau, Marc Suquet, Josep V. Planas, Rebeca Moreira, Antonio Figueras, Beatriz Novoa, Tomaso Patarnello, Luca Bargelloni

**Affiliations:** 1 Department of Comparative Biomedicine and Food Science, University of Padova, Legnaro, Padova, Italy; 2 Ifremer, UMR 6539 CNRS/UBO/IRD/Ifremer, Laboratoire des sciences de l’Environnement Marin (LEMAR), Plouzané, France; 3 Departament de Fisiologia i Immunologia, Facultat de Biologia, Universitat de Barcelona i Institut de Biomedicina de la Universitat de Barcelona, Barcelona, Spain; 4 Instituto de Investigaciones Marinas (IIM-CSIC), Vigo, Pontevedra, Spain; Shanghai Ocean University, CHINA

## Abstract

The king scallop *Pecten maximus* is a high valuable species of great interest in Europe for both fishery and aquaculture. Notably, there has been an increased investment to produce seed for enhancement programmes of wild scallop populations. However, hatchery production is a relatively new industry and it is still underdeveloped. Major hurdles are spawning control and gamete quality. In the present study, a total of 14 scallops were sampled in the bay of Brest (Brittany, France) to compare transcriptomic profiles of mature oocytes collected by spawning induction or by stripping. To reach such a goal, a microarray analysis was performed by using a custom 8x60K oligonucleotide microarray representing 45,488 unique scallop contigs. First we identified genes that were differentially expressed depending on oocyte quality, estimated as the potential to produce D-larvae. Secondly, we investigated the transcriptional features of both stripped and spawned oocytes. Genes coding for proteins involved in cytoskeletal dynamics, serine/threonine kinases signalling pathway, mRNA processing, response to DNA damage, apoptosis and cell-cycle appeared to be of crucial importance for both oocyte maturation and developmental competence. This study allowed us to dramatically increase the knowledge about transcriptional features of oocyte quality and maturation, as well as to propose for the first time putative molecular markers to solve a major bottleneck in scallop aquaculture.

## Introduction

The king scallop, *Pecten maximus* (Linnaeus, 1758), is a native European species of high economic value. Global production is based on both fisheries and aquaculture with 55,726 and 38 tons in the year 2014, respectively [1]. Despite the large gap between fishery and farming production, FAO statistics underestimate aquaculture output since it does not consider the amount of hatchery-produced seed employed in restocking programs that recently increased, notably in France [2–4]. To overcome bottlenecks in *P*. *Maximus* hatchery production, to date research has mainly focused on bivalve physiology under farm-specific conditions, (*e*.*g*. [5–8]). However, hatchery production of this species is still hampered by difficulties occurring in broodstock conditioning, larval rearing, and infectious disease management. Among these major hurdles, spawning control and gamete quality are the most important issues for broodstock conditioning and larval rearing.

In hatcheries, bivalve gametes are obtained by applying thermal shocks or by stripping mature breeders/spawners. Spawning success in *P*. *maximus* is not predictable, with frequent failures to induce gamete emission. This bottleneck cannot be overcome by stripping as scallop stripped oocytes appear unfertile due to the need for a maturation process along the genital ducts [9]. In the genera *Pecten* and *Crassostrea* [10–11], spawning induces meiosis exit from prophase I and germinal vesicle breakdown (GVBD), then oocytes are further blocked at the first metaphase (metaphase I). The release from metaphase I is naturally triggered by fertilization or can be artificially induced [12]. Scallop and oyster oocytes encounter two blockages during meiosis I, yet meiotic progression differs between these species. Naturally spawned oocytes of both genera are blocked at metaphase I and wait for fertilization to re-enter meiosis. In oyster, gametes stripped from ovaries are still at prophase I but their suspension in seawater permits GVBD and progression up to metaphase I, thus allowing fertilization [13]. In contrast, stripped and hydrated scallop oocytes remain blocked at prophase prior to GVBD and cannot be fertilized [14]. In *R*. *decussatus*, gene-expression profiling demonstrated that specific biological processes like cell-cycle, calcium regulation, and WNT signaling are likely associated with stripped egg infertility [15]. Such molecular determinants of gamete maturation processes remain to be investigated in pectinids.

High variability in fish and shellfish reproductive success has been shown to be partly attributable to gamete quality, sperm–egg interaction, and differential viability of genotypes [16–17]. Therefore, in the last decade the interest in gametes quality of marine species has substantially increased [18–22]. Gamete quality is influenced by both abiotic and biotic factors. Notably, food availability, nutritional quality, temperature, photoperiod and salinity are key aspects affecting oocyte development as well as pollutants and harmful microorganisms (*e*.*g*. [17, 23–24]). In addition, oocyte quality can be also affected by poor husbandry practices (*e*.*g*. broodstock conditioning) [25].

Oocyte quality in fish has been defined as the potential of oocytes to produce a viable progeny and can be measured by embryo development yields [26], corresponding in bivalves to D-larval yields, which is considered as the best descriptor of oocyte quality in several taxa. However, estimating egg quality through the assessment of developmental success is time-consuming and technically difficult.

Accordingly, the identification of predictive markers (*i*.*e*. oocyte features that can be quantitatively measured to predict the developmental rate of embryos) able to get over this issue might assume key importance [21]. Predictive markers of female gamete quality have been extensively studied in many freshwater and seawater fish species [17]. Some quality criteria are size, shape, transparency, chorion and coelomic fluid aspects, distribution and volume of lipid droplets and floatability rate [27]. In addition, recent studies demonstrated that transcriptomic and proteomic data might be associated to low or high quality eggs [19–20, 28–29]. However, an effective and simple proxy of gamete quality does not exist yet and it is still very difficult to accurately assess the quality of gametes prior to fertilization [21].

Compared with fish species, only a small panel of criteria is used to assess quality in bivalves, including gonad color [30], mean eggs size [23], oocyte organic matter and lipid content [23, 31]. Unfortunately, all these indicators did not consistently reflect the quality of gametes (*e*.*g*. [22]) and reliable parameters can be assessed only after fertilization (*i*.*e*. fertilization success, D-larval yields and survival). This highlights the complexity of predicting embryo development in molluscs, as already suggested in fish [21]. In this context, global transcriptional studies might help in understanding the complex molecular mechanisms underneath oocyte maturation and quality (*e*.*g*. [19, 32–33]).

In the present study, a total of 14 females were sampled in the bay of Brest (Brittany; France). For eight of them, mature oocytes were collected by spawning induction using thermal stress whereas oocytes from the six remaining females were collected through gamete stripping. Microarray analysis was then performed by using a custom oligonucleotide microarray. The two main objectives of the present work were (i) to investigate transcriptional features of scallop spawned oocytes in relation to gamete quality estimated via D-larval rates and (ii) to explore gene expression profiles characterizing released oocytes (REL) compared to ovarian oocytes obtained by stripping (STR). These analyses provided relevant information on transcriptional profiles putatively involved in egg fertility.

## Methods

### Ethics statement

The great scallop is not considered as an endangered or protected species in any international species catalogue, including the CITES list (www.cites.org) and it is not included in the list of species regulated by the EC Directive 2010/63/EU. Therefore, no specific authorization is required to work on scallop samples. The experiments were monitored and carried out by authorized staff to minimise the animal’s suffering.

### Biological samples and RNA isolation

At the beginning of their natural spawning period, adult scallops (mean weight±SD: 174±32g, mean length: 111±7mm) were caught from Pointe du chateau (Logonna-Daoulas, France, 48.334955, -4.317432). The scientific fishing of this species was provided by the Brittany prefect (authorization number 267/2014). Scallops were transferred to the experimental hatchery of Ifremer (Argenton, France) where they were conditioned for 1 month under suitable conditions for germ cells maturation. Briefly, scallops were placed in experimental raceways supplied with 1 μm-filtered running seawater at 17 ± 1.0°C and fed with a mixed diet of two microalgae (*Chaetoceros gracilis* and *Tisochrysis lutea*) at a daily ratio equal to 10 exp 9 cells of each algae species/scallop.

Released oocytes were obtained by thermal stimulation to induce spawning of females, consisting on exposure to alternate cycles of 18°C (20 minutes) and 23°C (1 hour) [34]. Once spawning was completed, the collected oocytes were filtered in a 20 μm sieve, to avoid self-fertilization. Oocytes from eight females were rinsed with iso-osmotic ammonium formate (3% w/v) to remove salt. A total of 20,000 oocytes were homogenized in 1,5 ml of Extract-all (Eurobio) and stored at -80°C for further transcriptomic analyses. Fertilization was then performed as described in [19]. Trochophores movement was estimate at 24h post fertilization using a CASA device, according to [35]. Then, the D-larval yield was assessed at 48h post fertilization (number of normal D-larvae/total number of oocytes) as described in [20].

In addition, gametes (20,000 oocytes per female) from six sexually mature females were dissected and oocytes were collected by “gamete stripping” as reported in [36]. About 20,000 oocytes from each female were harvested and stored as described above. The remaining stripped oocytes from each female were fertilized (as described above) and D-larval rate was registered.

RNA was isolated by following the Extract-all manufacturer instructions and combining the RNeasy Mini Kit (Qiagen) for the nucleic acid purification. A DNAse treatment was also carried out (Qiagen). Samples concentration was measured in a NanoDrop^®^ ND-1000 spectrophotometer and the RNA quality was assessed through the Bioanalyzer 2010 instrument (Agilent).

### Microarray experiments

The 8x60K microarray platform accommodating a total of 59,824 probes has been deposited in the GEO database (http://www.ncbi.nlm.nih.gov/geo/) under accession number GPL22720. It was designed in the context of the European project REPROSEED (FP 7-KBBE-2009-1-2-11) that funded the high throughput sequencing of several *P*. *Maximus* tissues. Details on the sequencing data, the resulting assembly and the microarray design were reported in S1 File, while the sequences of the 45,488 contigs successfully employed for the *P*. *maximus* DNA microarray platform design have been provided in S2 File.

At the time of data analysis, the annotation of each contig employed for the microarray design was performed again, by running blastx similarity searches (cut off e-value of <1.0 E-5) against the updated release of several protein databases. The best hits against UniProtKB/SwissProt high quality proteins (release 2016_10—November 02, 2016), *Danio rerio*, *Drosophila melanogaster*, *Homo sapiens*, *Gasterosteus aculeatus*, *Nematostella vectensis*, *Capitella teleta*, *Strongylocentrotus purpuratus*, *Lottia gigantea* and *Crassostrea gigas* available on Ensembl Genome Browser (release 82, September 2015) and Ensembl Metazoa (release 33, October 2016) provided at least one match for 31,579 (52.8%) out of the total amount of transcripts. The best blastx hit of each probe against all the selected protein databases is reported in S1 Table.

Probe sequences and other details on the microarray platform can be found in the GEO database (http://www.ncbi.nlm.nih.gov/geo/) under accession number GPL22720.

Microarray experiments were carried out on a total of 14 samples corresponding to stripped oocytes (n = 6) and spawned oocytes (n = 8). Sample labelling and hybridization were performed according to the Agilent One-Color Microarray-Based Gene Expression Analysis protocol with the Low Input Quick Amp Labelling kit. Briefly, for each sample, 100 ng of total RNA was linearly amplified and labelled with Cy3-dCTP. In order to verify the technical robustness of the microarray work-flow, a mixture of 10 different viral poly-adenylated RNAs (Agilent Spike-In Mix) was added to each RNA sample before amplification and labelling. Labelled cRNA was purified through the RNAeasy Mini Kit (Qiagen), and sample concentration and specific activity (pmol Cy3/mg cRNA) were measured in a NanoDropHND-1000 spectrophotometer. A total of 600 ng of labeled cRNA was prepared for fragmentation by adding 5 ml 10X Blocking Agent and 1 ml of pre-warmed (60°C) 25X Fragmentation Buffer, and finally diluted by addition with 25 ml 2X GE Hybridization buffer. Forty ml of hybridization solution was then dispensed in the array (a slide contained eight arrays). Slides were incubated for 17 h at 65°C in an Agilent hybridization oven, subsequently removed from the hybridization chamber, quickly submerged in GE Wash Buffer 1 to disassemble the slides and then washed in GE Wash Buffer 1 for approximately 1 minute followed by one additional wash in pre-warmed (37°C) GE Wash Buffer 2.

### Data acquisition, correction and normalization

Hybridized slides were scanned at 2μm resolution using an Agilent G2565BA DNA microarray scanner. Each slide was scanned two times at two different sensitivity levels: XDR Hi 100% and XDR Lo 10%. The two generated images were analysed together, data were extracted and background subtracted using the standard procedures provided in the Agilent Feature Extraction Software version 10.7.3.1. To evaluate goodness and reliability of spot intensity estimates the software returns a series of spot quality measures. All control features (positive, negative, etc.), except for Spike-in (Spike-in Viral RNAs), were excluded from subsequent analyses.

The fluorescence values were normalized by performing a quantile normalization in R statistical software. Statistical analyses were performed on 35,770 out of 59,824 probes with signal higher than background in at least 6 out of 14 target samples. A log base 2 transformation was applied to all expression values and finally the parametric Combat algorithm [37] was implemented in R in order to adjust for the known between-experiments batch effect (i.e. different microarray slides). Normalized data were deposited in GEO archive under accession number GSE90679.

### Data analysis

A T-test, implemented in TMeV, was used to identify differentially expressed probes between stripped and spawned oocytes. Only the differentially expressed probes showing a significant variation have been selected (Bonferroni-adjusted p-value <0.05; Fold Change (FC) > 1.5).

To identify the transcripts whose expression was positively or negatively associated with the D-larval rate, a PMT template matching analysis (TMeV) was carried out on log2 fluorescence values and D-larval rates of released oocytes, setting a threshold p-value of 0.05 and a minimum correlation value (R) of 0.7.

A more systematic, functional interpretation of significant genes was then obtained through enrichment analysis using the Database for Annotation, Visualization, and Integrated Discovery (DAVID) software [38]. “KEGG Pathway”, “Biological process” (BP), “Cellular component” (CC), “Molecular function” (MF) annotation categories were used by setting the gene count equal to 3 and the maximum p-value equal to 0.05. Because DAVID database contains functional annotation data for a limited number of species, it was necessary to link the scallop transcripts with sequence identifiers that could be recognized in DAVID. This process was accomplished using UniProtKB/SwissProt feature identifiers corresponding to each probe. These identifiers were used to define a “gene list” (*i*.*e*. significant probes) and a “background” (*i*.*e*. all the probes represented in the array) in the bioinformatic tool DAVID, corresponding to differentially transcribed scallop genes and to all the transcripts that were represented on the array, respectively.

## Results

### Hatching rates

**Fig 1:**
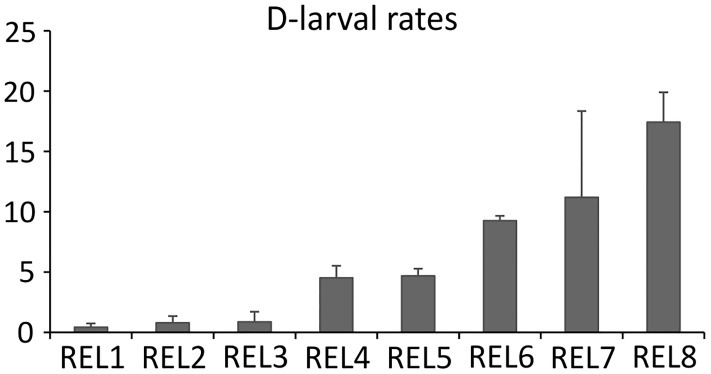
D-larval rates. Values of D-larval rates of released oocytes (REL) expressed as percentages of trochophore at 48 hpf on the total count of oocytes employed for the fertilization. Standard deviation refers to batch replicates (n = 3) as described in [20].

Thermal stimulation effectively induced gamete release in both males and females. At 48 hours post-fertilization, D-larval rates registered in each batch was in the expected range from 0.43% to 17.41% [39], depending on the female (Fig 1). Conversely, the fertilization of stripped oocytes did not produce any D-larvae.

### Correlation between gene expression profiles and D-larval rates

The PMT template matching analysis allowed the identification of a total of 1,904 probes (S2 Table) whose expression pattern was either positively (973) or negatively (931) correlated with D-larval rate values. Among these, a putative annotation against UniProtKB/SwissProt database was attained for 925 probes, corresponding to 848 unique proteins. The probes having the highest positive (R = 0.98) and negative (R = - 0.96) correlation factors did not have any match against the considered databases. The list of the putative protein identity of the most significantly correlated (R > 0.9) transcripts annotated against the protein database UniProtKB/Uniprot has been provided in Table 1.

**Table 1:** Transcripts with the highest correlation between gene expression and D-larval rates (R>0.9).

**Transcripts positively correlated with D-larval rate**	**R correlation**
Lactoylglutathione lyase (Q9CPU0)	0.96
Baculoviral IAP repeat-containing protein 6 (O88738)	0.96
Acetyl-coenzyme A synthetase, cytoplasmic (Q9QXG4)	0.95
Heparanase (Q9MYY0)	0.94
Phosphatidylinositol-binding clathrin assembly protein LAP (Q9VI75)	0.94
Transposable element Tcb2 transposase (Q04202)	0.94
Heparanase (Q9Y251)	0.94
Protein NRT1/ PTR FAMILY 8.1 (Q9M390)	0.94
Dual specificity testis-specific protein kinase 2 (Q924U5)	0.94
Protein FAM179B (Q6A070)	0.93
Heparan-sulfate 6-O-sulfotransferase 1 (O60243)	0.93
Importin-4 (Q8VI75)	0.93
E3 ubiquitin-protein ligase MIB2 (Q5ZIJ9)	0.93
Phosphatidylinositol-binding clathrin assembly protein (Q13492)	0.93
E3 ubiquitin-protein ligase RNF34 (Q6AYH3)	0.93
Androgen-induced gene 1 protein (Q9NVV5)	0.93
Protein RRNAD1 (Q96FB5)	0.93
Ras-related protein Rab-30 (Q923S9)	0.92
Mitogen-activated protein kinase kinase kinase 9 (Q3U1V8)	0.92
Anoctamin-7 (Q6IFT6)	0.92
Lactoylglutathione lyase (Q9CPU0)	0.92
Rho GTPase-activating protein 44 (Q5SSM3)	0.91
Insulin receptor substrate 2-A (Q9DF49)	0.91
E3 ubiquitin-protein ligase MSL2 (Q9HCI7)	0.91
Ig-like and fibronectin type-III domain-containing protein 1 (O18016)	0.91
Zinc finger protein 711 (A2ANX9)	0.91
Parkinson disease 7 domain-containing protein 1 (Q29RZ1)	0.91
Kinesin-like protein KIF14 (L0N7N1)	0.91
Calcium-binding mitochondrial carrier protein SCaMC-2 (Q5XH95)	0.91
Uroporphyrinogen-III synthase (P06174)	0.91
**Transcripts negatively correlated with D-larval rate**	**R correlation**
Ribonucleoside-diphosphate reductase small chain (P07201)	-0.95
Coiled-coil domain-containing protein 61 (Q08CF3)	-0.94
Serine/arginine-rich splicing factor 4 (Q8VE97)	-0.94
CWF19-like protein 1 (Q8AVL0)	-0.94
Tropomyosin-2 (P43689)	-0.93
Histone H1.2 (P15796)	-0.93
Retinol dehydrogenase 12 (Q96NR8)	-0.93
60S ribosomal protein L12 (P35979)	-0.93
Mitotic-spindle organizing protein 1 (Q0VFD6)	-0.92
Di-N-acetylchitobiase (Q01460)	-0.92
Ankyrin repeat domain-containing protein 30B (Q9BXX2)	-0.92
Large proline-rich protein BAG6 (A3KPW9)	-0.92
Protein Fer3 (Q9VGJ5)	-0.92
Target of EGR1 protein 1 (Q9D2E2)	-0.92
Glomulin (Q92990)	-0.92
F-box/LRR-repeat protein 15 (Q91W61)	-0.91
E3 ubiquitin-protein ligase TRIP12 (Q14669)	-0.91
RNA-directed DNA polymerase from mobile element jockey (P21329)	-0.91
Peptidyl-tRNA hydrolase 2, mitochondrial (Q8R2Y8)	-0.91
Probable tRNA pseudouridine synthase 2 (Q5XGG2)	-0.91
Protein slowmo (Q9V3U9)	-0.90
High affinity copper uptake protein 1 (Q8WNR0)	-0.90

Transcript names are those retrieved from UniProtKB/SwissProt database.

**Fig 2:**
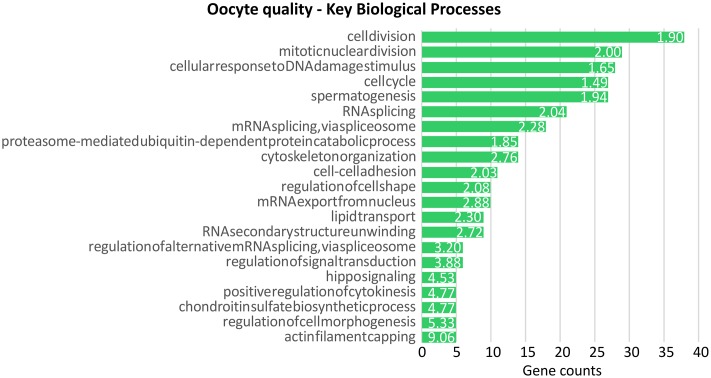
Enrichment analysis of transcripts significantly correlated with D-larval rates. Significant enriched BP_direct terms obtained through the enrichment analysis performed on the transcripts significantly correlated with D-larval rates. The green bars identify the number of the correlated genes belonging to the annotation term. Only terms with minimum gene counts of 5 were reported. Numbers beside the bars correspond to the Fold Enrichment reported for each term.

In order to investigate the main biological processes that most likely affect oocyte quality reflected by D-larval rates of each female, a functional enrichment analysis was performed by using the 811 UniProt accession numbers recognized in DAVID as gene list (S3 Table, Fig 2). Significantly enriched BP terms were “cell division” (Fold Enrichment FE: 1.89), “mitotic nuclear division” (FE: 1.99), “cytoskeleton organization” (FE: 2.75) and “RNA splicing” (FE: 2.03).

Among the significant genes participating in cell-cycle and cell division processes there were *Excision repair cross-complementation group 6-like* (ERCC6L; R = 0.89), *Anaphase promoting complex subunit 1* (APC1; R = 0.86), *Lymphoid-specific helicase* (LSH; R = 0.74), *Regulator of chromosome condensation* (RCC1), *BTB (POZ) domain containing protein 1* (RCBTB1; R = -0.75), and *Spindle and kinetochore associated complex subunit 2* (SKA2; R = -0.84). Positive correlated probes encoding transcripts involved in cytoskeleton regulation included *Spectrin alpha* and *Spectrin beta*, *Kinesin family member 14* (KIF14; R = 0.91), *Phosphatidylinositol-binding clathrin assembly protein LAP* (PICALM; R = 0.94), *Diaphanous-related formin 2* (DIAPH2; R = 0.73) and *Neurabin-1* (PPP1R9A; R = 0.87). For RNA processing, transcripts positively correlated with D-larval rates were *WT1-associated protein* (WTAP; R = 0.72), *Tudor domain-containing protein 1* (TDRD1; R = 0.79) and *Pumilio RNA-binding family member 1* (PUM1; R = 0.78), while several DEAD box proteins (DDX55, DDX19A, DDX39A, DDX39B and DEAD box protein UAP56) were negatively correlated to oocyte developmental competence. Furthermore, a large number of splicing factors was either negatively or positively associated to oocyte quality (*e*.*g*. *Splicing factor 4*, *Splicing factor 9G8*, *Splicing factor arginine/serine-rich 16*, *Splicing factor arginine/serine-rich 2*).

**Fig 3:**
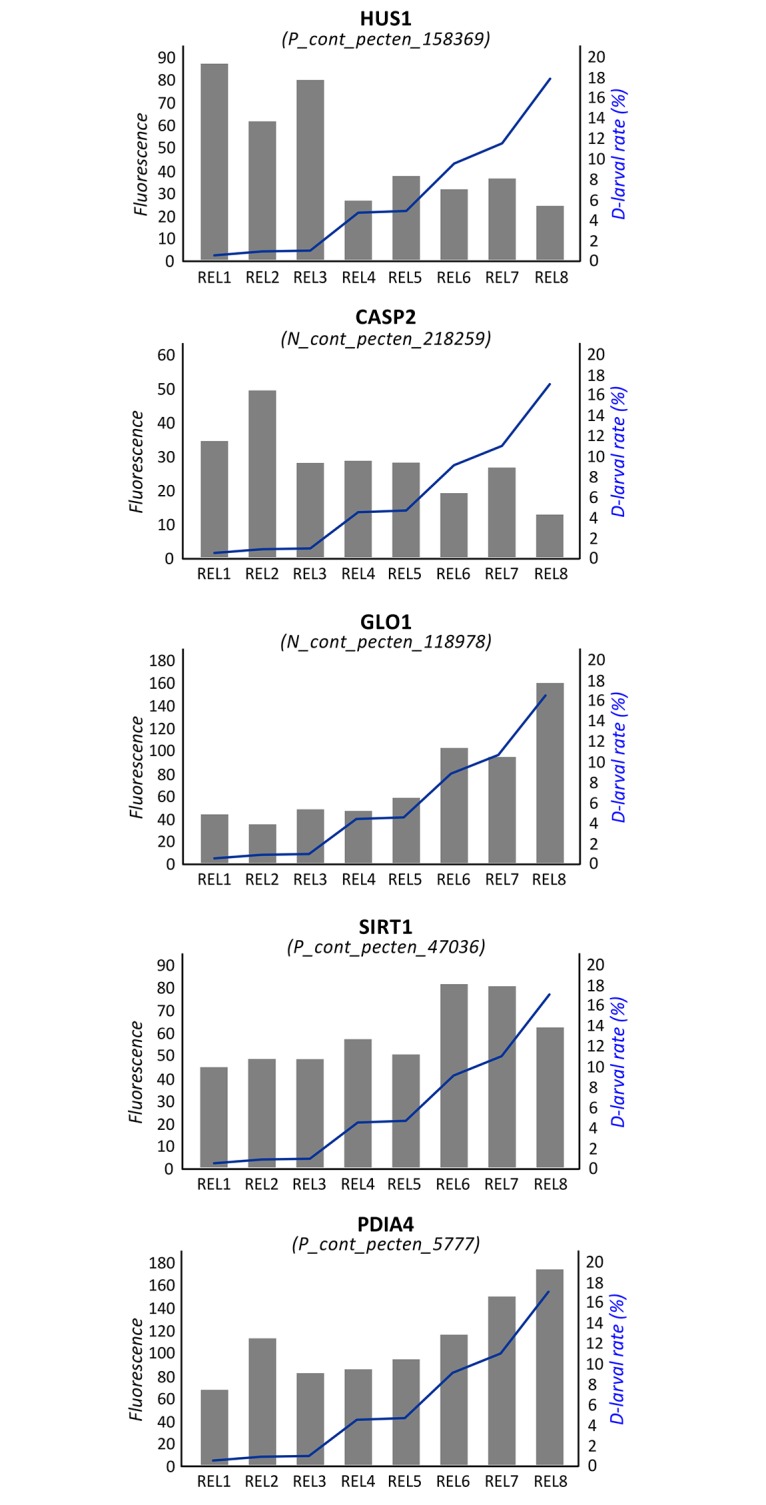
Correlation between gene expression and D-larval rates. Values of fluorescence reported for probes encoding HUS1, CASP2, GLO1, SIRT1 and PDIA4. Samples are reported in the x axis. Expression level (principal y axis) is expressed in terms normalized fluorescence. The D-larval rate is reported in term of percentage (secondary y axis) and described by a blue line.

An additional enriched BP term was “cellular response to DNA damage stimulus” (FE: 1.65). In fact, several significant genes were involved in facing DNA damage and regulating apoptosis. *Checkpoint protein HUS1* (see Fig 3), *Caspase 2* (CASP2; see Fig 3), *Growth arrest and DNA damage-inducible protein* (GADD45A), and *Peptidyl-tRNA hydrolase 2* (PTH2) were negatively associated to D-larval rates, while *Glyoxalase I* (GLO1; see Fig 3), *Regulator of telomere elongation helicase 1* (RTEL1), *Sirtuin 1* (SIRT1; see Fig 3) and the *Baculoviral IAP repeat-containing protein 6* (BIRC6) were more transcribed in oocytes with the highest developmental competence.

Probes encoding enzymes involved in energy producing processes were also highlighted: *Acetyl-coenzyme A synthetase* (ACECS), synthetizing Acetyl-CoA for the tricarboxylic acid (TCA) cycle, was highly correlated to oocyte quality (R = 0.95). Moreover, a similar transcriptional behaviour was evidenced for two enzymes driving the TCA cycle: *alpha-ketoglutarate dehydrogenase-like* and *putative malate dehydrogenase 1B*.

Full lists of significantly enriched GO terms and KEGG pathways are reported in S3 Table (gene count>3, p-value < 0.05).

### Transcriptional differences between stripped and spawned oocytes

**Fig 4:**
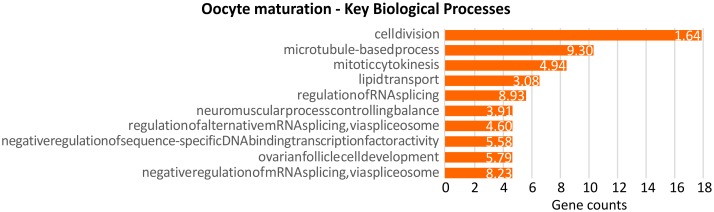
Enrichment analysis of DEGs between stripped and released oocytes. Significant enriched BP_direct terms obtained through the enrichment analysis performed on DEGs between STR and REL oocytes. The orange bars identify the number of the correlated genes belonging to the annotation term. Only terms with minimum gene counts of 5 were reported. Numbers beside the bars correspond to the Fold Enrichment reported for each term.

Pairwise comparison among stripped and spawned oocytes revealed a total of 1,682 probes differentially expressed (S4 Table). Among these, 652 and 1,030 probes were more expressed in REL and STR oocytes, respectively. Putative annotation against UniProtKB/SwissProt database was attained for 597 probes, corresponding to 546 unique proteins. Enrichment analysis, carried out by using 480 UniProtKB/SwissProt accession IDs recognized in DAVID database, evidenced the significant enrichment of 16 BP terms and 6 KEGG pathways (S5 Table, Fig 4). The most significantly enriched BP term was “microtubule-based process” (FE: 9.30), represented by several tubulin alpha isoforms, more expressed in STR oocytes. “Regulation of RNA splicing” was also enriched (FE: 8.93) with genes like *CDC-like kinase 2* (CLK2) and *SON DNA binding protein* (SON), both found more expressed in STR compared to REL oocytes. Likewise, the term “negative regulation of mRNA splicing, via spliceosome” (FE: 8.23), represented by splicing factors SRSF12 and SFSWAP, and *U2 small nuclear ribonucleoprotein auxiliary factor* (U2AF), was significantly overrepresented. The BP term “Ovarian follicle cell development” was also found significantly enriched (FE: 5.79), with *Beta-1*,*3-galactosyltransferase brn* and *Homeobox protein Cut* genes over-expressed in REL oocytes. Finally, among enriched BP, several terms related to cell division have been detected, such as “cell division” (FE: 1.64), and “mitotic cytokinesis” (FE: 4.94). Examples of probes involved in cell division and significantly more expressed in REL oocytes were those encoding *Cyclin-dependent kinases regulatory subunit 1* (CKS1), *Cyclin O* (CCNO), *Ubiquitin-conjugating enzyme E2-17 kDa* (UBE2D1) and ERCC6L. Conversely, *Cell division cycle 42* (CDC42) and *Protein MIS12 homolog* (MIS12) were more expressed in STR than REL oocytes. Noteworthy, additional transcripts more expressed in *P*. *maximus* STR oocytes were the egg yolk precursor *Vitellogenin-4* (VTG4; FC = 238), the *glycan binding Galectin-4* (GAL4, FC = 129), a *Fatty acid-binding protein* (FABP, FC = 90), the phosphotransferase *Arginine kinase* (AK, FC = 15), the detoxifying enzymes *Glutathione S-transferase sigma* (GSTS, FC = 46) and theta (GSTT, FC = 2.78), and the *Serotonin receptor 5-hydroxytryptamine receptor 4* (HTR4; FC = 2.94). Probes expressed at higher levels in REL oocytes were those coding for *nuclear protein Akirin-2* (AKIR2; FC = 9), *Diacylglycerol kinase eta* (DGKH; FC = 7.46) generating phosphatidic acid (PA), and *mitochondrial Isocitrate dehydrogenase [NAD] subunit beta* (IDH3B; FC = 1.76).

## Discussion

A key aspect deserving special attention is the generally low D-larval rates obtained from the fertilization of the eight females spawning, ranging from 0.4% to 17.4% (Fig 1). In bivalves, individual variability in oocyte quality is commonly observed [16, 40] representing a key factor in hatchery-based shellfish production. Notably, previous studies carried out in the great scallop demonstrated that D-larval rates achieved in hatchery conditions are particularly low [41] especially if compared to what experienced in other commercial bivalves such as in the Pacific oyster *C*. *gigas* [39]. In our study, broadly different performances were reported across scallops (Fig 1). Because both environmental and experimental conditions were kept uniform across animals, such variability was most likely due to the intrinsic quality of oocytes of each individual.

**Fig 5:**
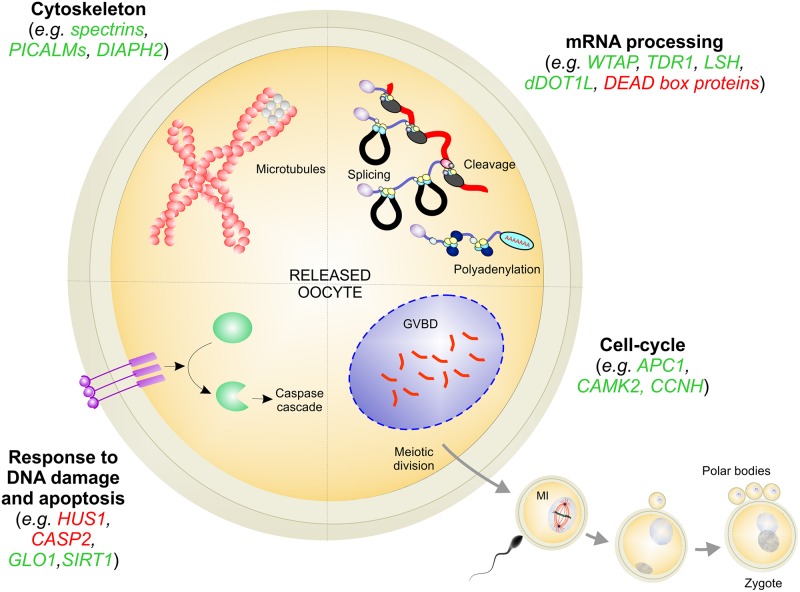
Main processes affecting oocyte quality. Genes positively and negatively correlated with D-larval rates were reported in green and in red colour, respectively.

The enrichment analysis carried out on the transcripts significantly correlated with hatching rates, pointing out several biological processes that most probably regulate the quality of scallop spawned oocytes and determine their fate (S3 Table). Overall, the expression level of genes involved in cytoskeletal dynamics, mRNA processing, DNA damage, apoptosis, and cell-cycle play an important role in assuring oocyte competence and sustaining the very first larval development in scallop (Fig 5). For the sake of clarity, these biological processes will be discussed separately.

### Cytoskeleton

Seven probes positively correlated to oocyte quality showed a high sequence similarity with alpha and beta spectrins. Spectrins act as actin crosslinking and molecular scaffolds and a few studies suggested that they may function globally in coordinating cytoskeletal functions within epithelial tissues during early embryo development (*e*.*g*. [42*–*43]).

A putative member of the kinesin superfamily of microtubule-associated motors (KIF14), highly correlated to D-larval rate, was demonstrated in *Xenopus* oocytes to be required for mitotic cytokinesis and to bind the central spindle [44]. Despite at a lower significance, additional kinesins, such as KIF6 and KIF2A, appeared to be more expressed in scallop oocytes having the highest hatching rate. Oocyte kinesin stocks might play a crucial role in coordinating interactions between actin and microtubule cytoskeleton of the scallop embryo, and thereby contribute to faithful cytokinesis occurring in the early developmental phases.

Furthermore, four probes encoding PICALM were positively correlated to oocytes competence. PICALMs are the major proteins recruiting clathrin to cell membranes at sites of coated-pit formation and clathrin-vesicle assembly, thus mediating endocytosis of plasma membrane receptors, channels, and transporters, as well as transmembrane proteins and various soluble macromolecules [45]. Besides PICALMs, the mRNA expression of two additional molecules involved in clathrin-mediated endocytosis was positive related to D-larvae yield: the enzyme Synaptojanin-1 and the accessory protein Epsin-2 (see the review [46]). Such multiple lines of evidence suggest two hypotheses on the importance of the endocytic complex in scallop REL oocytes. First, a higher abundance of these transcripts could better sustain endocytic processes, which provide nutrients and mediate cellular signalling during early larval development. Secondly, we hypothesize that these transcripts in *P*. *maximus* oocytes might be translated during the first life stages activating mitogenic signalling pathways of crucial importance for cell growth and differentiation.

Finally, DIAPH2 was also positively correlated to D-larval rate. DIA proteins are required for proper spindle formation, actin tubulin organization, cytokinesis and microtubule–kinetochore attachment, thus they are expected to play a crucial role in oocyte maturation. In the starfish *Asterina pectinifera* inhibition of DIAPH2 activity prevented cleavage furrow closure and resulted in polar body extrusion failure [47].

### mRNA processing

Once oocytes are released, meiosis resumption occurs and mRNA transcription is generally thought to cease [48]. However, translation of the stored pool of mRNAs continues throughout the final stages of meiosis [49] to synthetize proteins that are crucial to support oocyte maturation (meiotic maturation), as well as the phase prior to zygote-embryonic genome activation [50]. This evidence let us hypothesize that scallop spawned oocytes encoding the more “suitable” pool of molecules involved in the mRNA processing dynamics are those assuring higher D-larval rates.

WTAP, positively correlated with developmental competence, is a regulatory subunit of a methyl-transferase complex that has been demonstrated to act as a mRNA splicing regulator in human and mouse [51]. The importance of this gene during the early development has been proved in zebrafish embryo, where a WTAP knockdown caused marked tissue differentiation defects and increased apoptosis [52].

TDRD1, more expressed in oocytes with the highest D-larval rate, mediates the repression of transposable elements during meiosis in mice and zebrafish by acting via piwi-interacting RNA metabolic process [53]. The tudor gene was originally discovered in *D*. *melanogaster* in a screen for maternal factors that regulate embryonic development or fertility [54].

An important role is suggested also for epigenetic processes, as demonstrated by the observed transcriptional levels of LSH and histone H3-K79 methyl-transferase (dDOT1L), both positively correlated with developmental competence. LSH is a chromatin remodelling protein acting as epigenetic regulator [55]. Interestingly, a previous study reported that in mice LSH is essential for the establishment of homologous-chromosome synapsis, thus allowing the completion of meiosis [56]. Similarly, LSH disruption in mice caused global hypomethylation, developmental growth retardation, and a premature aging phenotype [57]. dDOT1L is a histone methyl-transferase specific for lysine 79 of histone H3 playing important roles in meiosis progression and supposed to be associated with chromosome deacetylation of mouse oocytes [58].

Additional probes whose expression changed according to D-larval rates were those coding for DEAD box proteins DDX55, DDX19A, DDX39A, DDX39B and DEAD box protein UAP56. DEAD box proteins regulate RNA secondary structure and are involved in translation initiation, ribosome assembly, RNA splicing and mRNA turnover in an ATP-dependent reaction. Thus, they are expected to play a central role in the oocyte, where stockpiled mRNAs are used to sustain oocyte maturation, fertilization and embryo development until the embryonic genome is activated. In the present study, DEAD box proteins were negatively correlated to D-larval rates. Despite the function of these proteins has been scarcely explored in bivalve oocytes (*e*.*g*. [59]), a previous study conducted in the Atlantic surf clam, *Spisula solidissima*, suggested that a DEAD box protein represses translation of maternal mRNA in early development [60]. Moreover, Minshall and co-workers demonstrated that the helicase activity may be attenuated during meiotic maturation, prior to cytoplasmic polyadenylation, allowing mRNA translation of key developmental proteins. Accordingly, the higher mRNA expression of DEAD box proteins in poor-quality REL oocytes might reflect stronger repression of translation and lower maturation level, compared to oocytes with higher D-larval rates.

### Response to DNA damage and apoptosis

Programmed cell death may lead to DNA fragmentation and oocyte degeneration [61], meaning poor oocyte quality and lower fertility in mammals [62–63]. Thus, apoptosis in oocytes has been considered a marker of oocyte quality and its capacity to develop into a viable embryo. There are several evidence supporting the idea that apoptosis in the oocyte can affect embryo quality because of the presence of maternal mRNAs stored in the oocyte that regulate the apoptotic mechanism [64–65].

In the present study, several transcripts correlated with oocyte quality were involved in cellular response to DNA damage stimuli. *Checkpoint protein HUS1* (Fig 3) and *DNA damage-binding protein 2* (DDB2) were negatively associated to D-larval rates. HUS1 is a component of the 9-1-1 cell-cycle checkpoint response complex. In *Drosophila* it plays a major role in homologous recombination DNA repair [66] and is essential for activation of the meiotic checkpoint [67]. Human DDB2 plays important roles in nucleotide excision repair and it is critical in deciding cell fate (apoptosis or arrest) upon DNA damage [68]. Thus, overexpression of HUS1 and DDB2 in poor quality oocytes suggests the presence of DNA damage. Conversely, RTEL1 and SIRT1 (Fig 3) were positively correlated with D-larval rates. RTEL1 is an ATP-dependent DNA helicase required to suppress inappropriate homologous recombination, thereby playing a central role in the protection of genome against instability. SIRT1 is a NAD-dependent deacetylase suggested to be a marker of oxidative stress and aging in mammals [69–70] and it has been demonstrated to protect oocytes against oxidative stress in mouse [71]. Similarly, in pigs, sirtuins are involved in cortical polarity and spindle organization and their inhibition adversely affects oocyte meiosis [72]. SIRT1 and GLO1 (Fig 3) have been also demonstrated to participate in the cellular pathways activated by the oocyte to counteract methylglyoxal (MG), a highly reactive dicarbonyl promoting AGE (advanced glycation end-products) accumulation and oxidative stress [73]. Here, GLO1 expression was highly correlated (R = 0.96) to scallop developmental competence (Fig 3).

Several transcripts that encode molecules regulating apoptosis were correlated to D-yield. The most interesting transcripts were those coding for Large proline-rich protein BAG6, CASP2, GADD45 alpha and BIRC6. BAG6 plays a role in protein folding and proteasomal degradation [74] and is involved in DNA damage-induced apoptosis. In *Xenopus* egg extracts, binding of apoptosis inducer factor Reaper to BAG6 promoted cytochrome c-mediated caspase activation leading to cell death [75]. CASP2 is crucial for oocyte apoptosis in the mouse and in *X*. *laevis* [76], since it induces apoptosis by releasing pro-apoptotic proteins from mitochondria. Evidence that BAG6 and CASP2 negatively correlated with D-larval rates (Fig 3) suggests that programmed cell death has likely a high incidence in oocytes with low developmental competence. This was also suggested in *R*. *decussatus* oocytes, showing a negative correlation between D-larval yield and Caspase 8 (CASP8) [20]. GADD45A is a stress-inducible nuclear protein involved in maintenance of genomic stability, senescence, apoptosis, DNA repair [77] and suppression of cell growth, and has a key role in active DNA demethylation that occurs in *Xenopus* oocytes [78]. The negative correlation between GADD45 expression and D-yields corroborates the hypothesis that oocytes with low developmental competence might exhibit elevated apoptosis. Conversely, BIRC6 was expressed at higher extent in oocytes with higher D-larval rates. BIRC6 is believed to inhibit apoptosis by targeting key cell-death proteins, thus a higher transcription in REL oocytes might be linked to protection from programmed cell death. Similarly, BIRC6 expression is crucial for embryo survival during bovine preimplantation embryo development [79].

### Cell-cycle

Several transcripts acting through cell-cycle were correlated to D-larval rates. APC1 was more expressed in oocytes with the highest D-larval rates. APC is an E3 ubiquitin-ligase essential for progression through meiosis since it promotes Cyclin B destruction and meiotic exit until fertilization occurs. Notably, APC has been recognized in mammals as a key molecular determinant of oocyte quality of direct relevance to reproductive performance [80]. APC is activated by the Ca2+ signal by a meiosis-specific mechanism via calcium/calmodulin-dependent kinase II (CAMK2) [81]. A putative scallop CAMK2 alpha chain was highly correlated with oocyte competence, thus reinforcing the hypothesis that also in scallop oocytes, as in model species [82], APC Ca-dependent signaling might enable meiosis progression.

Gene expression of a putative Cyclin H (CCNH) was lower in oocytes showing poor developmental competence compared to discrete-quality oocytes. Despite the functions of CCHN in meiosis have been poorly investigated, this evidence is in accordance with a previous study demonstrating that suppression of CCNH inhibits pig early meiotic resumption and maturation to MII [83].

Additional interesting genes significantly correlated to D-larval rates were *Heparanase* (HPSE), *Anoctamin-7* (ANO7), *Phospholipase C-beta-1* (PLCb1) and P*rotein disulfide-isomerase A4* (PDIA4; Fig 3). HPSE is an endoglycosidase that cleaves heparan sulfate, thus participating in degradation and remodeling of the extracellular matrix. Notably, HPSE supplementation resulted in approximately a two-fold increase in mouse embryo implantation rate in vivo [84]. Anoctamins have been proposed to be responsible for Ca2+-activated Cl− currents and in *Xenopus* oocytes these channels play a role in the fast block to polyspermy [85]. PLCb1 is a phosphoinositide-specific phospholipase having a role in resumption of meiosis in the mouse oocyte [86–87]. PDIs are chaperone molecules advantageous for the viability and immune protection of eggs and early embryos of Pacific oyster, and it is up-accumulated in good quality oyster oocytes [19]. Previous studies reported that PDIs are involved in oocyte development [88] and sperm–egg fusion at fertilization [89–90], suggesting that also sperm–egg interaction are crucial determinants in oocytes quality and developmental success.

### *P*. *maximus* oocyte maturation

Gene expression analysis and evaluation of DEGs between oocytes before and after spawning provides a first overview on transcriptome changes that are most likely correlated with scallop stripped oocyte infertility. Key biological processes affecting oocyte maturation were those regulating cell division, with several genes being more expressed in REL oocytes. Two interesting examples are CKS1 and CCNO. CKS1 encodes a protein that binds to cyclin-dependent kinases and regulates cell-cycle progression [91]. In *C*. *elegans*, CKSs were demonstrated to have an essential role in meiosis M phase exit [92]; mice lacking CKS2, a mammalian homolog of yeast CKS1, were viable but sterile due to failure of female germ cells to progress past the first meiotic metaphase [93]. Likewise, CCNO is a cyclin acting as upstream regulator of MPF and demonstrated to play an important role in mouse oocytes since the CCNO knockdown blocked meiosis resumption [94]. Thus, higher expression of transcripts encoding CKS1 and CCNO in REL oocytes, might suggest that higher synthesis of cell-cycle regulators is necessary for scallop oocyte maturation. Conversely, we found a few cell-cycle genes that were more expressed in immature oocytes compared to REL oocytes. This is the case of CDC42, a member of the Rho family of small guanosine triphosphatase proteins, playing pivotal roles in the establishment of mouse oocyte cellular polarity [95]. In mouse, CDC42 high-expression levels have been revealed in Germinal Vesicle (GV) stage oocytes (prophase I stage) and the expression decreased up to the 2-cell (2C) stage embryo [96].

Noteworthy, a few genes included in the enriched term “ovarian follicle cell development were more expressed in REL oocytes. The transcript encoding BRAINIAC protein (Beta-1,3-galactosyltransferase brn) was an interesting example. *D*. *melanogaster* BRAINIAC is a secreted protein produced by oocytes and its activity is needed in the germ line for proper organization of the follicle [97].

The probe showing the higher FC was the one encoding *Vitellogenin* (Vg-4), being more expressed in STR oocytes. Vitellogenins are large phospholipoglycoprotein precursors that are cleaved to generate yolk storage proteins traditionally regarded as the energy reserve for nourishment of the developing embryo [98]. Higher Vg expression in STR compared to REL oocytes likely reflects maturation stage. In fact, intra-gonadal oocytes arrested in prophase I, before being released and fertilized, undergo a period of vitellogenesis [99] that requires extensive Vg synthesis. In *M*. *galloprovincialis*, vitellogenesis seems to stop before spawning, since full-grown intra-gonadal oocytes do not express Vg mRNA [100].

Nutrients and energy reserves are key factors in supporting oocyte development. Accordingly, a *Fatty acid-binding protein* and a putative AK were more expressed in scallop STR oocytes. In zebrafish a FA-binding protein (FABP3) has been demonstrated to be most abundant immediately prior to and during the vitellogenic stage of oocyte development and to decrease during the oocyte growth phase, being nearly undetectable in matured oocytes [101]. Likewise, the ovarian expression of AK, related to provision of energy, was higher in a penaeid shrimp during pre-vitellogenic stages and decreases in mature oocytes [102].

A similar expression pattern also for a *Glutathione peroxidase* (GPx), which is expressed at a higher extent in STR oocytes compared to REL group. GPx may function in protecting maturating oocytes against peroxidation [102]. Besides GPx, two glutathione-dependent enzymes showed higher expression in STR oocytes: GSTS and GSTT. The relation between GST and oocyte maturation has never been studied in bivalves, however in humans a negative correlation between GSTT mRNA and cumulus-oocytes complexes maturity has been demonstrated [103].

Finally, an interesting gene showing a differential expression between STR and REL oocytes was the excitatory *Serotonin receptor HTR4* (more expressed in STR group). Serotonin is a major neurotransmitter that triggers spawning and oocyte germinal vesicle breakdown (GVBD) in bivalve molluscs [104–106]. Expression levels of HTR4 might be low in released oocytes because spawning was already occurred, and high in stripped oocytes, since HT receptors are still engaged in sustaining meiosis progression prior to spawning.

## Conclusions

In this study, a new species-specific microarray platform for great scallop has been employed to identify differentially expressed genes in relation to oocyte quality and to investigate the transcriptional features of both stripped and spawned scallop oocytes.

To date, most of the studies on egg quality and development have been conducted in model species, therefore it seems too early to hypothesize specific functions of the majority of transcripts expressed in scallop oocytes. The lack of scallop genome and, in general, of high quality bivalve genomes also hinders a full comprehension of transcriptomic data. However, sequence similarity searches against model species allowed us to infer putative functions of expressed transcripts, thus allowing the identification of candidate transcriptomic markers of oocytes quality, such as CASP2 and PDIA4 (Fig 3), both positively associated to female gametes quality also in other bivalve species [19–20]. The identification of generic mechanisms shared by evolutionary distant species is of special interest, since in-depth investigation of these pathways in a single species could lead to important knowledge applicable to aquaculture practices of a large number of species [21]. At the same time, cross-species conserved pathways might provide more reliable markers, which could be used to develop cost-effective tools for rapid assessment of oocyte quality. In this perspective, the validation of these biomarkers in additional hatchery-based productions might be of crucial interest.

## Supporting information

S1 File Pecten maximus transcriptome assembly. (DOCX)

S2 File Sequence of the contigs employed for the DNA microarray platform. (FASTA)

S1 Table Blastx best hit of each probe represented in the microarray platform. (XLSX)

S2 Table Correlation between gene expression and D-larval rate. (XLSX)

S3 Table Enrichment analysis of transcripts correlated with D-larval rate. (XLSX)

S4 Table DEGs between stripped and spawned oocytes. (XLSX)

S5 Table Enrichment analysis of DEGs between stripped and spawned oocytes. (XLSX)
