# Supplementary material for: Transcriptomic features of Pecten maximus oocyte quality and maturation
Source: PLoS One. 2017 Mar 2;12(3):e0172805. doi: 10.1371/journal.pone.0172805 (PMC5333834; doi:10.1371/journal.pone.0172805)
Supplement: S1 File — (DOCX) [file pone.0172805.s001.docx]

***Pecten maximus* transcriptome assembly**

Several sequencing data previously obtained in the context of the European project REPROSEED (FP 7-KBBE-2009-1-2-11) were used to build the transcriptome scaffold that served for the microarray platform design. How the sequencing reads have been obtained and which procedures have been followed to construct the assembly will be explained below.

Sampling and cDNA libraries construction

Several scallop specimens (adult tissues, larvae, hemocytes and oocytes) were obtained thanks to SCALPRO hatchery (Norway), the *Consejo Superior de Investigaciones Científicas* (CSIC, Spain) and the *Institut Français de Recherche pour l'Exploitation de la Mer* (IFREMER, France). All samples were stored in RNAlater at -80°C until the RNA purification. RNA was isolated with RNeasy Mini Kit (Qiagen), following the manufacturer instructions and a DNAse treatment (Qiagen) was carried out. Concentration and purity of RNA were measured using a *NanoDrop ND1000* spectrophotometer (NanoDrop Technologies). The RNA quality was assessed through the Bioanalyzer 2010 instrument (Agilent).

Non normalized libraries for RNA sequencing experiments were prepared by using Truseq RNA sample prep Kit (Illumina) following the manufacture’s instruction and sequencing was carried out in the Illumina Hi-Seq 2000 by running a multiplexed lane 2x100bp paired ends (BGI Tech, Shenzhen, China). All the Illumina reads were analyzed with FastaQC software in order to assay the sequences quality.

A full-length-enriched double stranded cDNA synthesis was performed using MINT cDNA synthesis kit (Evrogen, Moscow, Russia) according to manufacturer's protocol, and was subsequently purified using the QIAquick PCR Purification Kit (Qiagen USA, Valencia, CA). The amplified cDNA was normalized using Trimmer kit (Evrogen, Moscow, Russia) to minimize differences in representation of transcripts. The normalized 454-library was sequenced with a Roche 454 GS FLX sequencer using the Titanium chemistry (Genomic unit, CCiT-UB, Barcelona, Spain). Finally, a sequences quality report was obtained through the 454 Software Release 2.6.

The number of sequenced reads per library are reported in Table A.

Reads assembly

In order to build a *P. maximus* transcriptome scaffold, a mixed strategy was preferred. Different assemblers were employed according to the sequences origin (454 or Illumina). The 454-sequences were assembled through MIRA3 (default parameters), the reads from each of the Illumina libraries were separately assembled by using *CLC Genomic Workbench 5* (default parameters). Finally, to reduce the redundancy, a merge of all the obtained contigs was carried out through CAP3 (default parameters). The contigs not merged in CAP3 were joined to all the meta-contigs obtaining a final transcriptome scaffold of 226,824 contigs. The reads assembly strategy was summarized in Table A.

| **SEQUENCING DATA and FIRST ASSEMBLY** | | | | | |
| --- | --- | --- | --- | --- | --- |
| **Sample** | **Technology** | **Total reads** | | **SRA accession number** | **Contigs obtained** |
| Stripped oocytes | *Illumina* | 66,454,364 | | SRR5062040 | 101,627 (CLC) |
| Released oocytes | *Illumina* | 84,121,748 | | SRR5062041 | 46,961 (CLC) |
| In vivo/vitro control hemocytes | *Illumina* | 67,643,592 | | SRR1009240 | 59,067 (CLC) |
| In vivo stimulated hemocytes | *Illumina* | 70,855,070 | | SRR1009241 | 58,512 (CLC) |
| In vitro stimulated hemocytes | *Illumina* | 77,946,012 | | SRR1009242 | 46,734 (CLC) |
| Mix of adult tissues, larvae, hemocytes, oocytes | *Roche 454* | 1,105,257 | | SRR5059346 | 88,552 (MIRA3) |
| **CAP3 SUPER-ASSEMBLY** | | | | | |
| N° of contigs in CAP3 input | | | 401,453 | | |
| N° of meta-contigs produced by CAP3 | | | 44,346 | | |
| N° of singletons produced by CAP3 | | | 182,478 | | |
| **Complete scaffold** | | | **226,824** | | |

**Table A. Sequencing and Assembly strategy**. The number of reads per library, their SRA accession numbers and the assembly strategy were specified.

Functional annotation

A functional annotation of the assembled transcriptome was attained through blastx similarity searches conducted against UniProtKB/SwissProt database (release 2012_10 – October 31, 2012) and several protein databases available on Ensembl Genome Browser (release 68): *Homo sapiens*, *Danio rerio*, *Drosophila melanogaster*, *Caenorhabtidis elegans*, *Ciona intestinalis*, *Strongylocentrotus purpuratus*, *Gasterosteus aculeatus*, *Daphnia pulex* and *Nematostella vectensis*. In addition, to improve the number of annotated contigs, blastx searches against two molluscs databases were attempted: oysterDB (*Crassostrea gigas)* and *Lottia gigantea V1.0.* Alignments with an E-value of at most 1 E-5 were considered significant and only the best hit per contig was taken into account. Blastx results were summarized in Table B.

| **Database** | **N° of match** | **N° of not redundant match** |
| --- | --- | --- |
| *Danio rerio* Ensembl | 37,948 | 12,721 |
| *Homo sapiens* Ensembl | 35,812 | 14,031 |
| UniProKB/SwissProt | 38,350 | 18,420 |
| *Drosophila melanogaster* Ensembl | 28,642 | 7,644 |
| *Nematostella vectensis* Ensembl | 37,312 | 10,938 |
| *Caenorhabtidis elegans* Ensembl | 24,625 | 6,555 |
| *Ciona intestinalis* Ensembl | 30,998 | 8,173 |
| *Strongylocentrotus purpuratus* Ensembl | 68,955 | 12,331 |
| *Gasterosteus aculeatus* Ensembl | 35,692 | 11,376 |
| *Daphnia pulex* Ensembl | 31,576 | 8,115 |
| *Lottia gigantea* V1.0 | 45,498 | 12,640 |
| *Crassostrea gigas* OysterDB | 50,021 | 13,524 |

**Table B. Blastx annotation**. The number of contigs having a match against each database are reported. The number of unique match (i.e. the total number of genes in each database showing a significant sequence similarity with at least one contig) were also specified.

Microarray design

All databases used for the annotation step were considered to reduce the redundancy in annotated contigs. A total of 31,849 contigs found non-redundant (with a unique annotation) in at least one reference database have been considered for microarray design. Putative sense-strand orientation was inferred from the matching protein-coding gene in reference data bases. For 671 contigs that showed ambiguous orientation two probes with opposite orientations (sense and antisense) were designed. For the remaining 31,178 contigs with putatively un-ambiguous orientation a single (sense) probe was designed.

Since the microarray format could accommodate approximately 60,000 probes, non-annotated contigs that showed the highest expression in hemocytes based on RNA-seq data were included in the microarray design. In total 13,740 non-annotated contigs were added, and for each of them, two probes with opposite orientation (sense and antisense) were designed. Probe design was carried out using the Agilent eArray interface (https://earray.chem.agilent.com/earray/), which applies proprietary prediction algorithms to design 60-mer probes. A total of 59,824 out of 60,000 probes were successfully obtained, representing 45,488 different *P. maximus* contigs.
